# Growth Assessment of Under-Five Children of Employed and Unemployed Mothers of Etawah District, Uttar Pradesh: A Cross-Sectional Study

**DOI:** 10.7759/cureus.48035

**Published:** 2023-10-31

**Authors:** Pradip Kumar, Sandip Kumar, Mohit Mishra, Kirti Jaiswal, Prem Prakash Bharati, RS Yadav, PK Jain, Vineet Kumar, Mamta Yadav

**Affiliations:** 1 Community Medicine, Uttar Pradesh University of Medical Sciences, Etawah, IND; 2 Physiology, Uttar Pradesh University of Medical Sciences, Etawah, IND; 3 Botany, Karm Kshetra Post Graduate (K.K. P.G. College, Etawah, IND; 4 Research, Private Practice, Etawah, IND

**Keywords:** under five, unemployed mothers, employed mothers, children, growth

## Abstract

Introduction

The under-five age group is crucial because the health profile of this age group will have a huge effect on the future development of the nation. Early infancy is marked by several distinct developmental characteristics. Progress in each domain of childhood development is used to track a child's development.

Objectives

The objectives of the present study were to assess and compare the growth of under-five children of employed and unemployed mothers in the Etawah District of Uttar Pradesh, India.

Material and methods

A community-based cross-sectional survey was carried out in Etawah district’s urban and rural areas between January 2021 and June 2022. A total of 200 mothers with children under the age of five were recruited using the purposive sampling method. To gather pertinent information, a semi-structured, pre-tested, interviewer-administered questionnaire was used.

Results

In the present study on the comparison of the growth of children among employed and unemployed mothers, it shows that 48 children (50.5%) with a weight between 10.5 and 15 kg were of employed mothers, while 52 children weighing less than 10.5kg were of unemployed mothers (p<0.001). Forty-four children (57.1%) with a chest circumference of more than 48 cm were of employed mothers, while 26 children (78.8%) with a chest circumference of less than 45 cm were of unemployed mothers (p = 0.001).

Conclusion

The present study indicates that statistically significant differences were found in age-appropriate gain in weight and chest circumference, which was higher among the children of employed mothers in comparison to children of unemployed mothers. There was no statistically significant difference in age-appropriate gain in height, head circumference, or mid-upper arm circumference among the children of employed mothers and unemployed mothers.

## Introduction

Children are the backbone of every country, and their well-being is a top priority for the government. The morbidity profile of the under-five age group is vital because it will have a substantial impact on the growth of the nation as a whole. Early infancy is marked by several distinct growth characteristics. During this time, children begin to develop significant communication abilities. Progress in each domain of childhood growth is used to track a child's growth. Changes in childhood growth are caused by the combination of maturation and learning processes. Both mother and child health are impacted by maternal work. The level of income and childcare practices are two of the main ways that maternal employment affects a child's nutrition [1]. The amount of money a woman makes and controls is crucial in supporting the budget for food for her family, her children, and herself [2]. On the other hand, employment that requires the mother's absence generally results in partial weaning or termination of nursing, making it impossible to supervise the child's nutrition and care [3].

Children under the age of five grow more rapidly when their mothers are employed. According to a study, moms who work in offices have much fewer underweight and wasted kids than those who work as laborers, farmers, or housewives. Stunting in children is also strongly correlated with the mother's work. The risk of becoming stunted, however, is not as great as the chance of being underweight and wasting away [4]. India has almost 1.4 billion citizens, making it the most populous nation in the world [5]. The National Family Health Survey (NFHS)-5 indicates that India's levels of stunting (height for age) are still intolerable. Children under the age of five were stunted in 35.5% (39.7% Uttar Pradesh) and were underweight (weight for height) in 19.3% (17.3% Uttar Pradesh) between 2019 and 2021. India is ranked 116th out of 174 countries in the human capital index [6]. Mothers who have had more schooling are more likely to have children who have grown more rapidly, but this relationship is not universal [7-9]. According to studies, children of employed women have a higher rate of childhood mortality than children of unemployed women [10].

The impact of maternal employment on children's growth, nutrition, and behavior has been the subject of several studies across the globe, although there aren't many studies done in India. Research on the impact of maternal employment on the growth of children under the age of five years is few. Participants in this study come from both urban and rural locations, and it aims to determine whether there are any systemic differences between children of moms who are employed and those who are not. As a result, the findings of this study will strengthen knowledge and promote policy creation to conduct intervention programs for the improvement of the nutritional status of children under the age of five. The findings of this study will also assist healthcare program designers, parents or guardians, physicians, and all other stakeholders in emphasizing the nutritional condition of children for working moms and women who are not working.

## Materials and methods

A community-based cross-sectional survey was carried out in Etawah district's urban and rural areas between January 2021 and June 2022. A total of 200 mothers (100 employed and 100 unemployed) with children under five years old were recruited using the purposive sampling method. To gather pertinent information, a semi-structured, pre-tested, and interviewer-administered questionnaire was used. In this study, employed mothers were those who, at the time of data collection, held regular work with a specified pay (Rs 1000/month) and a job length of at least six months. Data were collected by either house visits or from the working sites of mothers. In the event that the mother was unavailable when the data were collected, we telephonically contacted them to schedule an appointment before the data was collected.

Sample size

The sample size was determined by using a single population proportion formula by taking the prevalence of children under five years who were wasted as 12.1% as per NFHS 4 [11]. Assuming a 5% margin of error (absolute) and a 95% confidence interval, the sample size came out to be 163. Assuming a 10% non-response rate, the sample size came out to be 180. After rounding off, the final sample size was 200.

Eligibility criteria

In this study, we included 100 women with children under the age of five who were working, as well as 100 mothers without formal employment. The unemployed mothers were recruited through a door-to-door survey. In a family, if more than one eligible mother was present, the mother of the youngest child was included in the study. Employed mothers with at least one child under the age of five were recruited from schools, banks, and government offices. If a mother had more than one child under five years of age, then the youngest child was included in the study. The participants were explained in detail about the aim and objectives of the study in their native language. Before each interview and measurement, the mothers of the children enrolled in the study were asked for their written informed consent to participate in the study. The data were collected using a questionnaire, and the anthropometric measurements of children were done with proper precautions. The weight measurement was done using an electronic weight scale (the Virgo-9811B model), and the height measurements were done using a stadiometer. The closest units of measurement for height and weight were 0.01 kg and 1 cm, respectively. Additionally, scale indicators were checked against zero readings before and after weighing each child, in addition to instrument calibration. In accordance with WHO guidelines, anthropometric indices, Z-scores for height for age (WAZ), weight for height (WHZ), and mid-upper arm circumference (MUAC), were calculated [12].

Ethical consideration

Ethical clearance for the present study was obtained from the Institutional Ethics Committee, Uttar Pradesh University of Medical Sciences, Saifai, Ettawah, Uttar Pradesh, India (ethical clearance no. 168/2020-21). 

Statistical analysis

The data were entered into a Microsoft Excel spreadsheet (Microsoft Corp., Redmond, WA) and analyzed using IBM SPSS software version 24.0 (IBM Corp., Armonk, NY). Continuous variables were presented as means with standard deviations, and associations were considered statistically significant at a p-value threshold of 0.05.

## Results

A total of 100 participants were recruited in the employed group and 100 participants in the unemployed group after applying the inclusion and exclusion criteria to the children of employed and jobless mothers; no participants were lost to follow-up. Table 1 shows participants' sociodemographic information and reveals that the age group of 12-24 months constituted 101 children (50.5%) and accounted for the majority of the study participants' children.

**Table 1:** Distribution of the study participants based on their sociodemographic characteristics (n=200) OBC: Other Backward Castes; SC: Scheduled Castes; ST: Scheduled Tribes

Variables	Frequency	Percentage (%)
Age group (in months)		
12-24	101	50.5
25-36	59	29.5
37-48	40	20.0
Gender of the index child		
Male	102	51.0
Female	98	49.0
Religion		
Hindu	171	85.5
Muslim	27	13.5
Others	02	1.0
Category		
General	68	34.0
OBC	104	52.0
SC/ST	28	14.0
Educational qualification of the participants		
Illiterate	19	9.5
Up to intermediate education	70	35.0
Up to postgraduate qualification	111	55.5
Socio-economic status		
Upper class	41	20.5
Middle class	44	22.0
Lower class	115	57.5
Family type		
Nuclear family	77	38.5
Joint family	64	32.0
Three generational family	59	29.5

The participants in the study had an average age of 28.25 ± 10.67 months. The gender-wise distribution of children shows 102 males (51.0%) and 98 females (49.0%); the majority of the study subjects were Hindus (n=171, 85.5%). The category-wise distribution shows that most of the study participants were from Other Backward Castes (OBC) (n=104, 52.0%), followed by 68 from the general category (34.0%) and 28 from Scheduled Castes (SC) and Scheduled Tribes (ST) (14.0%). The majority of study participants (n=111, 55.5%) were educated up to the postgraduate level, and 19 (9.5%) were illiterate. Most participants (n=115, 57.5%) belonged to the lower socioeconomic class, followed by 44 from the middle class (22.0), and 41 from the upper socioeconomic class (20.5%), according to the modified BG Prasad socioeconomic scale.

Most of the children (n=180, 90%) had a birth weight of more than 2500 grams, while 95 of the children (47.5%) had a present weight between 10.5 and 15 kg. At the time of the study, 100 children (50.0%) had a height between 75.0 and 87.5 cm. The MUAC of 158 children (79.0%) was more than 12.5 cm, and nine children (3.0%) had MUAC less than 11.5 cm at the time of the study. The chest circumference of 90 children (45.0%) was 45.0-48.0 cm, and the head circumference of 160 children (80.0%) was 45.0-49.0 cm (Table 2).

**Table 2:** Distribution of study participants based on their growth assessment (n=200)

Variables	Frequency (n)	Percentage (%)
Birth weight		
More/less than 2500 gm	20	10.0
More than 2500gm	180	90.0
Present weight		
Less than 10.5 kg	88	44.0
10.5-15 kg	95	47.5
More than 15 kg	17	8.5
Present height		
Less than 75 cm	21	10.5
75-87.5 cm	100	50.0
More than 87.5 cm	79	39.5
Mid-upper arm circumference		
Less than 11.5 cm	09	3.0
11.5-12.5 cm	33	16.5
More than 12.5 cm	158	79.0
Chest circumference		
Less than 45 cm	33	16.5
45-48 cm	90	45.0
More than 48 cm	77	38.5
Head circumference		
Less than 45 cm	24	12.0
45-49 cm	160	80.0
More than 49 cm	16	8.0

On comparing the growth of children among employed and unemployed mothers, it was seen that 50.5% of children (n=48) with a weight between 10.5 and 15 kg and 94.1% of children (n=16) with a weight greater than 15 kg had employed mothers. Whereas 59.1% of children (n=52) with a weight less than 10.5 kg were of unemployed mothers. This difference in age-appropriate weight increase in children of employed and unemployed mothers was statistically significant (p<0.001). Most of the children of employed mothers had chest circumferences of 45-48 cm (n=49) and more than 48 cm (n=44), while the chest circumferences of 26 children of unemployed mothers were less than 45 cm. There was a statistically significant difference (p=0.001) in the chest circumference of children of employed and unemployed mothers. Head circumference, MUAC, and height of children did not show any statistically significant association with the employment of mothers (Table 3).

**Table 3:** Comparison of growth between under-five children of employed and unemployed mothers (n=200) ^*^Chi-square test; ^#^Fischer's exact test

Variables	Children of employed mothers (N=100) n (%)	Children of unemployed mothers (N=100) n (%)	P-value
Height (in cm)			
Less than 75	13 (61.0)	08 (39.0)	0.19*
75-87.5	44 (44.0)	56 (56.0)	
More than 87.5	43 (54.4)	36 (45.6)	
Mean ± SD	87.61 ±11.84	84.66 ±7.64	
Weight (in kg)			
Less than 10.5	36 (40.9)	52 (59.1)	<0.001^#^
10.5-15	48 (50.5)	47 (49.5)	
More than 15	16 (94.1)	01 (5.9)	
Mean ± SD	12.04 ± 3.36	10.92 ±1.70	
Head circumference (in cm)			
Less than 45	11 (45.8)	13 (54.2)	0.812*
45-49	80 (50.0)	80 (50.0)	
More than 49	09 (56.2)	07 (43.8)	
Mean ± SD	47.43 ±2.42	46.91 ±2.18	
Chest circumference (in cm)			
Less than 45	07 (21.2)	26 (78.8)	0.001*
45-48	49 (54.4)	41 (45.6)	
More than 48	44 (57.1)	33 (42.9)	
Mean ± SD	49.12 ±4.29	47.23 ±3.31	
Mid-upper arm circumference (in cm)			
Less than 11.5	03 (33.3)	06 (66.7)	0.2^#^
11.5-12.5	13 (39.3)	20 (60.7)	
12.5 or more	84 (53.1)	74 (46.9)	
Mean ± SD	13.62 ±1.25	13.17 ±1.03	

## Discussion

The current study shows that the majority of participants (50.5%) were between the ages of 12 and 24 months, 29.5% were between the ages of 25 and 36 months, and 20% were between the ages of 37 and 48 months. The mean age of the study participants was 28.25 ± 10.67 months. Most participants (51% of both groups combined) were male, followed by females at 49.0%. The majority of the study subjects in both groups were Hindus (85.5%), followed by Muslims (27%), and 2% percent were from other religions. Most of the study participants belonged to Other Backward Castes (52%), followed by 34% in the general category and 14% to the Scheduled Castes and Scheduled Tribes. In a study done by Hurissa et al. (2020) [13] on 254 children aged 0-59 months, an assessment of the effect of maternal employment on the nutritional status of children under five in Ethiopia shows similar findings to the present study, with the age group of children in the study group being as follows: six to 12 months: 89 (35%), 12-24 months: 111 (43.6%), and 24-59 months: 54 (21.4%), respectively, and the majority 103 (40.7%) of mothers were Muslims. In a study by Tadesse et al. (2019) [14], 113 working mothers and 445 unemployed mothers were compared. Among mothers who were employed, 60 (53.1%) and 160 (39.4%) of the respondents identified as Muslims, whereas 319 (71.7%) and 126 (28.3%), respectively, identified as Christians. Ayub et al. (2020) [15] revealed that the majority of them (77.3%) were Hindus and the remaining 22.7% were Muslims.

In a study by Nankinga et al. (2019) [16], the bulk of the children (59%) were between 24 and 59 months old, and (60%) were born weighing 2.5 kilograms or more. In a study by Rastogi et al. (2014) [17] titled “Does Maternal Employment Matter in Relation to Child Nutritional Status in Indian Metropolitan Cities?”, the results indicate that among children aged under one year (17.5%), between one and 12 years (21.1%), between 24 and 47 years (41.7%), and more than 47 months (20.6%), the majority (53.4% of children) were male and the minority (46.6%) were female.

The present study demonstrates that the weights of children in the employed and unemployed groups are significantly different (p<0.001). In a study, Eshete et al. (2017) [18] reported that 31 (9.8%) of the children were wasted, of which 8.8% and 10.8% were children of employed and unemployed mothers, respectively, and that 12.1% of the children were underweight. The findings of this study are consistent with the findings of the current study. Maternal employment was also linked to underweight but not wasting, stunting child feeding index (adjusted odds ratio (AOR)=0.16; 95% CI: 0.04-0.86). In a study conducted by Issaka et al. [19] in two Kassena-Nankana districts, children aged six to 24 months were underweight, and some feeding practices were related to the work level of the mother. This study contradicts the findings of the current study because the populations, data-gathering methods, sample sizes, etc. are different. Anderson et al. (2002) [20] observed that two of the most striking changes in the American family over the past several decades have been the rise in female employment and the rise in overweight children.

A study conducted by Rastogi et al. (2014) [17] showed a higher risk of children being underweight is linked to maternal employment in the service sector among the poorest sections of cities and employment in the agriculture/labor sector among the wealthier sections. The present study shows that there is a statistically significant (p<0.05) difference in chest circumference for the employed and unemployed groups, respectively. In a study, Hurissa et al. (2020) [13] indicated that anthropometric measurements with a higher MUAC value showed that children of working mothers had better growth status. The MUAC was 12.5 cm for 151 of the kids (57.2%), while 90-95 percentile height for age was measured for 50% of the kids. The findings of this study confirm those of the current study. Shroff et al. (2011) [2] reported that babies of mothers who participated more in household decision-making tended to be less underweight and wasted. According to these findings, enhancing maternal financial and decision-making autonomy might have a favorable effect on growth outcomes. In a study conducted by Sultan et al. (2014) [21], it was found that children of working and nonworking mothers, respectively, had a prevalence of stunting (stunted+severely stunted) of 63.8% and 77.1%, respectively. Among the children of working and non-working moms, the prevalence of being thin (stunted+severely thin) was 91.9% and 57.5%, respectively. Stunting prevalence was shown to be significantly correlated with the job level of mothers.

In another study conducted by Rastogi et al. (2014) [17], a higher risk of children being underweight is linked to maternal employment in the service sector among the poorest sections of cities and employment in the agriculture/labor sector among the richer sections. The study comes to the conclusion that motherly employment has a significant negative impact on children's development in the wealthier areas of cities but not on children in the poorer areas.

Limitations

The study has limited generalizability due to a small sample size. As the study period was short, it was unable to measure all the characteristics of growth in the study subjects. We did not collect data on fever, respiratory infections, diarrhea, worm infestations, nutrition/diet, and other chronic conditions, which could have been possible confounders in the study.

## Conclusions

The current study shows that the children of employed mothers fared better in terms of age-appropriate weight gain and chest circumference than the children of unemployed mothers, with a statistically significant difference. Children of employed mothers and unemployed mothers show similar age-appropriate growth in height, head circumference, and mid-upper arm circumference. Head circumference, mid-arm circumference, and height did not show any statistically significant association with employment.
